# Effects of quantitative marinating on meat quality, biogenic amines, and flavor compounds in crayfish meat

**DOI:** 10.3389/fnut.2025.1573987

**Published:** 2025-04-10

**Authors:** Kelang Kang, Fan Zhang, Fuhua Fu, Jie Ouyang, Yingjuan Wei, Shuhua Lin, Cheng Jiang, Meijuan Yu, Hui Yang

**Affiliations:** ^1^Agricultural Products Processing Institute, Hunan Academy of Agricultural Sciences, Changsha, China; ^2^Yuelushan Laboratory, Changsha, China

**Keywords:** crayfish meat, quantitative marinating, texture, flavor compounds, biogenic amine

## Abstract

Stewing is a traditional processing method, commonly used for crayfish meat (*Procambarus clarkii*). In this study, we used a novel method called quantitative marinating (QM) to reduce industrial waste during crayfish meat processing. The taste, flavor, and aroma of crayfish meat processed by boiling (CON), stewing (SG), and QM were investigated. The results showed that crayfish meat in both SG and QM had higher L* and b* values (*P* < 0.05). Crayfish meat subjected to QM exhibited significantly greater hardness, gumminess, and chewiness than SG (*P* < 0.05), which was associated with tightly packed muscle fibers, as observed via scanning electron microscopy. Both QM and SG exhibited lower bitterness and astringency compared with CON, as tested by electronic tongue. A total of 25 types of FAAs content showed significant changes in QM and SG (*P* < 0.05), with the umami amino acids and total amino acids in QM increased by 19.47 and 52.97%, respectively, compared with SG. The results of flavor 5′-nucleotides showed that GMP, AMP, and IMP in QM increased by 72.87, 48.78 and 51.98% compared with SG, respectively (*P* < 0.05). Headspace-gas chromatography-ion mobility spectrometry (HS-GC-IMS) identified 31 compounds, with QM having more volatile compounds such as anethole, linalool, and 1-octanol than SG. The levels of biogenic amines of tryptamine, phenethylamine, and histamine in QM decreased significantly compared with SG (*P* < 0.05). In conclusion, QM significantly improved the meat color, texture profile and taste-related qualities of crayfish meat while reducing the biogenic amines in crayfish meat.

## 1 Introduction

The red swamp crayfish, a member of the crustacean family, is native to the lakes and rivers of the southern United States and northern Mexico. This species is highly adaptable to various environments and has become one of the most widely introduced freshwater species due to its notable economic value (1). It was introduced to China in the 1930s for its culinary value and has become a delicacy in many Chinese restaurants since (2, 3). In 2022, China produced approximately 2.89 million tons of crayfish, accounting for over 70% of the global supply (4). Current research on the red swamp crayfish primarily focuses on biological invasion (5), aquatic and ecological environment protection (4), and breeding and raising techniques (6). However, research on the flavor characteristics of crayfish remains limited.

Different cooking methods can impart diverse flavors and textures to meat, resulting in protein denaturation that enhances digestion and absorption (7). Crayfish meat, classified as white meat, is a good source of high-quality protein, with a tender texture, low fat content, and high protein content (8). As an aquatic animal, crayfish inherently contain aldehydes, lipids, and other compounds that contribute to its off-flavors (9). Therefore, processing methods are crucial for transforming flavor and enhancing the taste of crayfish. Traditional cooking methods for crayfish typically involve frying or stewing (10), both of which cook the crayfish at high temperatures. Deep-frying often results in the accumulation of lipid peroxides and increased fat intake, which may be associated with hypertension, cardiovascular issues, and obesity (11). Stewing refers to boiling crayfish with seasonings such as salt, cayenne pepper, lemon, garlic, bay leaf, etc. However, prolonged stewing can lead to higher biogenic amine content and residues of nitrites, as well as reduced chewiness of the crayfish. Furthermore, stewing is associated with low energy efficiency and the waste of spices (12). Therefore, developing novel strategies for producing healthier, tastier, and more energy-efficient processing methods for crayfish remains important.

Flavor predominantly influences consumers’ decisions when purchasing meat products, and people prefer both tender and flavorful (13). Tenderness is a critical component in meat quality and is influenced by many factors (14–16). Hence, determining the optimal processing procedure to get a desirable tenderness is challenging. Inappropriate processing duration, either too long or too short, can negatively affect the structure and integrity of muscle fibers and alter meat flavor development (17). Therefore, both tenderness and flavor should be evaluated to attain the desired properties of meat products. The flavor of meat products is influenced by various components, including free amino acids, 5′-nucleotides, and volatile substances (18, 19). Previous studies have shown that frying crayfish at different temperatures produces various volatile flavor compounds, with 170°C yielding the highest content of these compounds (10). Additionally, different heat treatments alter the free amino acid composition in beef, resulting in different sensory evaluations (20). Moreover, Xu et al. (3) utilized electronic tongue and headspace gas chromatography-ion mobility spectrometry (HS-GC-IMS) technology to assess taste differences in crayfish raised under different conditions (3). Thus, studying the impact of various processing methods on the flavor of meat products requires comprehensive analysis using multiple flavor and aroma evaluation techniques, which is crucial for guiding the development of meat products.

**FIGURE 1:**
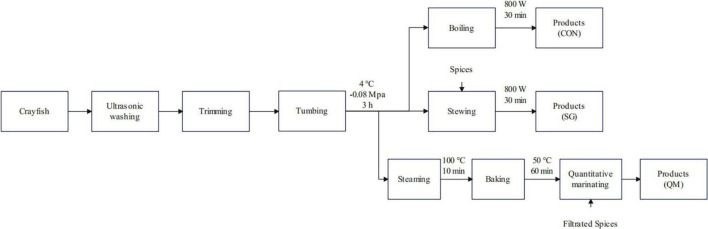
Flow diagrams of crayfish meat by CON, SG, and QM. CON, crayfish boiled in pure water; SG, stewing; QM, quantitative marinating.

In this study, we used a novel method called quantitative marinating to process the crayfish meat, aiming to reduce industrial waste during the processing of crayfish meat and verify if the quantitative marinating could improve the taste quality of crayfish meat (Figure 1). The physicochemical and flavor characteristics of crayfish meat proceeded by CON, SG and QM were investigated, including color, texture, microstructure of myofiber, and particularly, flavor and aroma compounds, as well as biogenic amines. These findings provide theoretical guidance for future research and processing methods of crayfish meat.

## 2 Materials and methods

### 2.1 Materials

Crayfish (*Procambarus clarkii*) tails and spices were purchased from a commercial company and used as experimental material. Crayfish of similar size, weighing 30 ± 2 g, were selected to ensure uniformity in sample weight (10). After removing the tail meat, it was washed with pure water and dried with absorbent paper before sample preparation. Ten different Chinese traditional spices were used, including mountain pepper (10 g), anise (7.5 g), fennel (6 g), amomum tsaoko (20 g), bay leaves (10 g), chili pepper (30 g), nutmeg (30 g), licorice (60 g), angelica dahurica (30 g), and kaempferiae (30 g). Spices were mixed and ground to powder. After that, place them in a spice bag and add 3 L of pure water. Maintain boiling for 1 h, until the volume of water reduces to 600 mL, then filter to obtain the spices marinade.

### 2.2 Sample pretreatment

The cooking method and process have been standardized prior to sample preparation. The cooking conditions were as follows: Control group (CON): all crayfish meat was boiled in pure water on an 800 W induction cooker, heating to 100°C and maintaining for 30 min. Stewing group (SG): the crayfish tails and spices were placed into water and stewed for 30 min on an 800 W induction cooker, heating to 100°C and maintaining for 30 min. Quantitative marinating group (QM): steaming in 100°C for 10 min and baking crayfish tail in 50°C for 60 min, then the mixed crayfish tail with filtered spices marinade. The spices added in stewed soup and in marinade were the same, calculated on a gram-per-gram basis of crayfish tail meat (Figure 1).

### 2.3 Analysis methods

#### 2.3.1 Determination of color

The color of crayfish meat was measured with a chromaticity meter (CR-400, Konica Minolta, Japan). Prior to use, a white calibration plate was used to calibrate the instrument. L* indicates the degree of brightness, a* value represents greenness and the redness, and b* value represents the change in color from yellow to blue.

#### 2.3.2 Texture profile analysis (TPA)

The TPA was examined following the condition described by Li et al. (21). Texture profile including the hardness, adhesiveness, cohesiveness, resilience, gumminess and chewiness were measured by a TMS-Pilot Food Texture Analyzer, and calculated by TPA program inside (Food Technology Corporation, Stering, VA, United States). TPA probe model was P50, diameter = 50 mm, and parameters were set with pretest speed = 120 mm/min, test speed = 60 mm/min, posttest speed = 120 mm/min, interval time = 5 s, trigger force = 5 g and compression ratio = 50%. First and second hardness are the maximum forces when the probe presses the meat sections for the first and second time, respectively.

#### 2.3.3 Scanning electron microscope

Cubes of crayfish tail meat (1 mm * 5 mm * 5 mm) were cut perpendicular to the muscle fiber and fixed with glutaraldehyde buffer solution for 4 h. Then meat was eluted with 10, 30, 50, 70, 80, 90, and 100% alcohol for 10 min, respectively. After freeze-drying for 24 h and 4 Pa, the meat samples were sprayed with gold (1.5 kV, 30 mA, 2 min), and the microstructure of the muscle was observed using scanning electron microscopy (JSM-5410, Jeol, Tokyo, Japan) at an acceleration voltage of 20 kV (22).

#### 2.3.4 Determination using electronic tongue

The e-tongue analysis was performed using the e-tongue system (TS-5000Z, Japan, INSENT). Five grams of crayfish tail meat were weighed, minced, and placed into a centrifuge tube. Twenty milliliters of distilled water preheated to 37°C were added, and the mixture was vortexed for 30 min. An additional 20 mL of distilled water was then added, followed by another 30-min vortexing. The mixture was centrifuged at 3,000 rpm for 10 min, and 35 mL of the supernatant was collected for the e-tongue test. Before testing each sample, the sensor was cleaned with a solution containing 30 mM KCl and 0.3 mM tartaric acid. The e-tongue, equipped with seven sensors, provided eight response values for each sample.

#### 2.3.5 Free amino acid content

Free amino acids in crayfish tail meat were analyzed according to a modified protocol (23). Two grams of crayfish meat were extracted by homogenization with 15 mL of 5% trichloroacetic acid for 1 min. Supernatant was obtained after centrifuged at 9,000 r/min for 15 min, 4°C. The pH of supernatant was adjusted to pH 6.5 with KOH and distilled to 10 mL. Then an amino acid analyzer L-8900 (Hitachi, Tokyo) was used to analyze free amino acid content (sample injection volume: 100 μL; wavelength; 570 nm, 440 nm). The results were expressed in mg/100 g of dry matter.

The typical umami amino acids (UFAAs) were calculated as the total of Asp and Glu. Sweet amino acids (SFAAs) were calculated as the total of Thr, Ser, Gly, Ala, Arg and Pro. Bitter amino acids (BFAAs) were calculated as the total of Val, Met, Ile, Leu, Phe, Tyr, Lys, and His.

#### 2.3.6 Quantitation of 5′-nucleotides analysis

Five grams of crayfish meat was homogenized centrifuged, and get supernatant were used for quantitation of nucleotide content. The 5′-nucleotides, including guanosine monophosphate (GMP), inosine monophosphate (IMP), and adenosine monophosphate (AMP), were extracted and analyzed using a HPLC system (UltiMate 3000, Thermo Fisher Scientific Co., Ltd., Massachusetts, United States), according to the method modified from Jin et al. (24). The parameters were set as follows: A chromatographic column (Acclaim PolarAdvantage Il C18, 50 mm × 4.6 mm, 3 μm); column temperature, 30°C; mobile phase A of methanol; mobile phase B of 20 mM KH_2_PO_4_-K_2_HPO_4_ buffer solution (v/v = 1:1, pH = 5.8); flow rate, 1 mL/min; 0% A and 100% B for 0-6 min, 8% A and 92% B for 7-14 min, 35% A and 65% B for 15-20 min, and 0% A and 100% B for 21-23 min, respectively.

#### 2.3.7 Volatile compounds

The volatile compounds of crayfish tail meat were performed by a Headspace-gas chromatography-ion mobility spectrometry (FlavourSpec^®^, Dortmund, Germany). For short, put 1 g of crayfish tail meat sample in headspace vial. Automatic headspace sampling was used with a sampling volume of 500 μL, an incubation time of 10 min at an incubation temperature of 50°C. Then nitrogen (99.99 %) was transport medium gas, and GC-IMS measure parameters were as follows: flow rates: 2 mL/min for 2 min, linear increase to 100 mL/min for 18 min. The total running time was 20 min, and column temperature was 55°C. After elution and separation at 40°C, nitrogen (99.99%) with a flow rate of 150 mL/min was used as the drift gas for IMS, under temperature of 45°C. Data from the HS-GC-IMS system were analyzed using LAV processing software. Use the *Reporter* plugin to compare the spectrum differences between samples; use the *Gallery Plot* plugin to compare the VOCs to identify differences in volatile organic compounds between different samples. The built-in NIST and IMS databases in LAV were used for qualitative analysis of substances.

#### 2.3.8 Biogenic amines

Six common biogenic amines (BAs), including tryptamine, phenethylamine, putrescine, cadaverine, histamine, tyramine, and spermidine were measured in 5 g samples using high-pressure liquid chromatography (HPLC) on a Shimadzu Prominence 20A System (Kyoto, Japan). BAs standards were purchased from Sigma-Aldrich (Shanghai, China). The derivatization using dansyl-chloride followed the method of Silbande et al. (25). Detection of the chromatographic peaks was performed with a UV detector (SPD-20A, Shimadzu) set at a wavelength of 254 nm.

### 2.4 Data analysis

According to the principles of randomized block design, each treatment group was subjected to three parallel experiments, with data presented as the mean ± standard deviation. Statistical data analysis was performed using SPSS 21.0 software. One-way analysis of variance and Duncan’s multiple comparison tests were conducted for each group. *P*-value < 0.05 was considered the criterion for significant differences. After statistical data analysis, Graph-pad Prism 8 software was used to draw bar graphs.

## 3 Results and discussion

### 3.1 Color, texture, and microstructure

**FIGURE 2:**
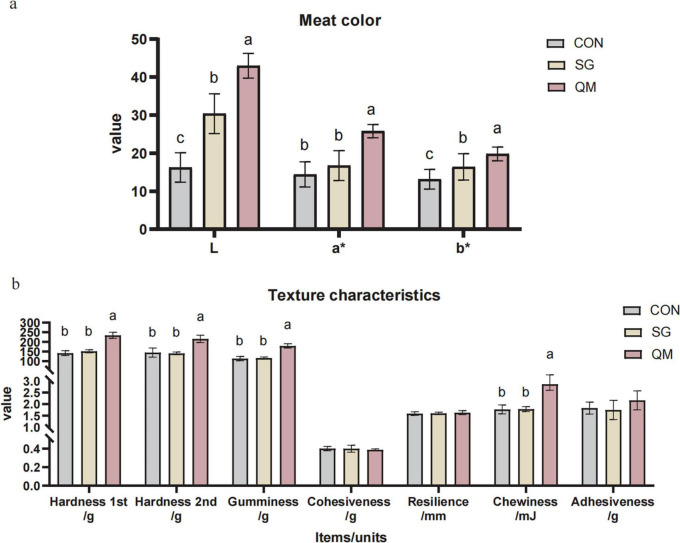
Meat color and texture profiles of crayfish meat processed by different methods. **(a)** Meat color. L, lightness; a*, redness; b*, yellowness. **(b)** Texture profiles. Each value is expressed as mean ± SD (*n* = 9). The different lowercase letters indicate significant differences (*P* < 0.05).

The meat color and texture in crayfish of each group are shown in Figure 2a and Supplementary Table 1. Stewing and quantitative marinating significantly affected the meat color, with the crayfish meat in QM showing the highest *L*, a* and b* values (*P* < 0.05). Stewing resulted in higher *L* and b* values in crayfish meat compared with CON (*P* < 0.05). The color of cooked meat is an important indicator of its quality, taste, flavor, and safety (26, 27). When crustaceans are cooked, the myoglobins responsible for the red color denature, leading to a characteristic bright red-orange hue (28). This color change is indicative of the myoglobin content, including deoxygenated and oxygenated myoglobin, which reflects the freshness of crayfish prior to cooking (29). Compared with CON, the L* of crayfish in the SG was significantly higher (30.4±5.19 vs. 16.25±3.87, *P* < 0.05). Following stewing, the *a* and *b* values of the crayfish meat increased by 15.9 and 24.5%, respectively. The SG and QM crayfish meat demonstrated increased brightness, redness, and yellowness values, with the QM group showed the highest values. In this research, spices may have contributed to the differences in crayfish meat color. For instance, chili peppers, widely used as natural colorants and flavoring agents in the food industry, and amomum tsaoko, which contains polyphenols and carotenoids in its brown shell, may contribute to a brownish-red coloration in the meat (30, 31). Stewing and QM processing methods may enhance the color-imparting effects of spice components, such as capsanthin, on shrimp meat.

The textural characteristics of processed meat products are critical to their quality, as they significantly influence the mouth feel experienced by consumers (32). Multiple factors affect meat texture, including the tightness of intramuscular connective tissue, thermal denaturation of muscle proteins and myofiber structure. Therefore, identifying an appropriate temperature for crayfish meat is important for texture quality. In this study, we evaluated meat texture index of different processing methods (Figure 2b and Supplementary Table 2). The results show that with the same boiling power and time, the stewing did not change any texture indices of crayfish meat compared with CON (*P* > 0.05). Compared to the SG group, the hardness (1st and 2nd), gumminess, and chewiness of crayfish meat in the QM group increased by 54.9, 54.6, 53, and 60%, respectively (*P* < 0.05). The primary distinction between the CON and SG groups is whether use of spices, while differences between the SG and QM groups are heat processing methods and spice pre-treatment application. A comparison of the CON and SG groups suggests that the use of spices does not significantly affect the texture characteristics. Therefore, it could be referred that the combination of steaming and low-temperature roasting in the QM group has pronounced impact on texture. To date, previous studies have linked heat treatment with modifications in the meat structure and its components. For instance, García-Segovia et al. (33) found that when cooking beef muscles for 1 h at 60∼80°C, the perimysium and endomysium become granular at 60°C and gelatinized at 80°C, and the cooking temperature significantly affects shear force (33). Jiang et al. (34) evaluated the impact of different cooking times on meat texture of crayfish meat, and the results showed that meat cooked at 93-95°C for 5 min had the highest hardness (34). Frying is also a widely used method for processing crayfish meat. When using an air-frying method at 190°C for 10 min, the hardness reached 353.41 N (10). These results are consistent with our findings. However, both stewing and QM did not have a significant effect on cohesiveness, resilience, and adhesiveness, compared with CON (*P >* 0.05). Cohesiveness is a measure of the degree of difficulty in breaking down the samples internal, and adhesiveness is an indicator of the protein hydrolysis index (35, 36). In present study, there was no significant difference observed in both SG and QM, compared with CON, which possibly means that the thermal treatments in both SG and QM methods completely denatured the proteins. Future research is necessary to elucidate the mechanisms by which the two processing methods and various spices influence the texture characteristics of crayfish meat.

**FIGURE 3:**
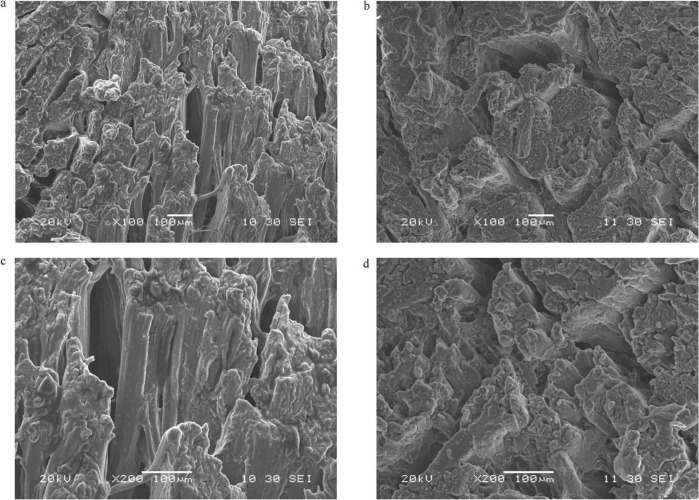
Scanning electron microscope images of crayfish meat. **(a)** Myofiber structure after stewing processing, 100×. **(b)** Myofiber structure after quantitative marinating processing, 100×. **(c)** Myofiber structure after stewing processing, 200×. **(d)** Myofiber structure after quantitative marinating processing, 200×.

Following the meat texture analysis, the muscle fiber structure was investigated using SEM. The microstructure of crayfish meat provides insights into the observed differences in texture indices. The quantitative marinating processing resulted in less disintegration of muscle fibers, with more complete cross-sectional areas and regularly arranged, tightly packed muscle fibers compared with the stewing process (Figure 3). Boiling and stewing for 30 min may apply excessive pressure to the muscle fibers, resulting in a loose structure. In contrast, the tighter connection of adjacent myofibers in the QM contributed to the observed increases in hardness, gumminess, and chewiness.

### 3.2 Electronic tongue

**FIGURE 4:**
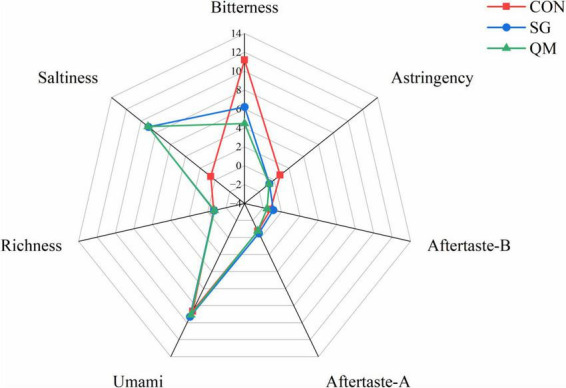
Taste radar graph of different processing methods. Aftertaste-B, aftertaste of bitterness; Aftertaste-A, aftertaste of astringency. Each value is expressed as mean ± SD (*n* = 9). The different lowercase letters indicate significant differences (*P* < 0.05).

To qualitatively evaluate flavor quality of crayfish meat, an E-tongue equipped with seven sensors was used to calculate taste activity values. The richness, umami, saltiness, bitterness, astringency, aftertaste bitterness, and aftertaste astringency of the crayfish meat samples were detected (Figure 4). Compared with CON, meat in SG and QM showed similar taste activity values, due to the same spices used during processing. Notably, both stewing and quantitative marinating with spices decreased bitterness and astringency and increased saltiness compared with the CON. These two processing methods imparted a unique flavor to the crayfish and reduced unpleasant tastes. To determine which method is superior, the composition of flavor compounds was further analyzed.

### 3.3 Free amino acids (FAAs) and derivatives

The concentration of FAAs is commonly used as an indicator of the eating quality and flavor of meat products. FAAs and peptides are precursors to the formation of some aroma compounds (37). Twenty-nine types of FAAs were identified in crayfish meat, as shown in Table 1. A total of 25 FAAs changed significantly (*P* < 0.05) in both SG and QM. The most abundant FAAs in QM meat were ornithine (295.83 ± 28.88 mg/100 g), an indicator of freshness of aquatic products (38), and alanine (273.75 ± 15.77 mg/100 g), which is positively correlated with sweetness (39). Alanine can be converted to acetaldehyde, which has a sweet taste through Strecker degradation (40). Both ornithine and alanine levels in QM were significantly higher than SG and CON (*P* < 0.05).

**TABLE 1:** Content of free amoni acids and derivatives in crayfish meats treated with three cooking methods (mg/100 g).

Item	Abbreviation	CON	SG	SBG	*P*-value
Phospho-serine	P-Ser	2.48±0.30^c^	3.34±0.26^b^	3.97±0.10^a^	0.001
Taurine	Tau	37.07±0.44^a^	3.42±0.19^c^	8.45±0.35^b^	<0.001
Aspartic acid	Asp	1.65±0.11^c^	8.37±0.65^b^	12.78±0.33^a^	<0.001
Threonine	Thr	11.18±0.84^a^	12.37±0.87^a^	8.71±0.30^b^	0.002
Serine	Ser	9.47±0.48^a^	4.03±0.32^b^	4.08±1.00^b^	<0.001
Glutamine	Glu	2.33±0.12^b^	28.67±2.37^a^	31.47±1.15^a^	<0.001
Glycine	Gly	44.60±2.22^c^	65.89±5.39^b^	146.47±10.48^a^	<0.001
Alanine	Ala	132.04±5.72^b^	112.63±9.11^b^	273.75±15.77^a^	<0.001
Citrulline	Cit	1.32±0.29^c^	116.40±10.04^b^	216.86±34.31^a^	<0.001
α-Aminobutyric acid	a-ABA	0.33±0.12^b^	0.47±0.09^b^	5.59±0.68^a^	<0.001
Valine	Val	18.51±1.31^b^	24.57±5.95^b^	31.80±1.35^a^	0.012
Methionine	Met	15.03±1.72^a^	9.42±0.76^b^	7.31±1.30^b^	0.001
cystathionine	Cysthi	0.62±0.15	0.53±0.22	0.53±0.06	0.735
Isoleucine	Ile	7.11±0.67^b^	11.68±0.87^a^	10.24±0.67^a^	0.001
Leucine	Leu	20.41±0.98^b^	28.66±2.14^a^	30.95±1.40^a^	<0.001
Tyrosine	Tyr	13.82±2.15^b^	19.66±1.31^a^	1.30±0.26^c^	<0.001
Phenylalanine	Phe	5.43±0.40^c^	14.48±1.17^a^	10.91±1.08^b^	<0.001
β-Alanine	b-Ala	1.31±0.31	1.25±0.12	1.59±0.15	0.194
β-aminoisobutyric acid	b-AiBA	3.23±0.10^a^	2.56±0.22^b^	1.13±0.16^c^	<0.001
γ-Aminobutyric acid	g-ABA	1.43±0.20	1.43±0.13	1.75±0.21	0.126
2-aminoethanol	EOHNH2	1.51±0.32^b^	1.67±0.10^b^	28.05±2.16^a^	<0.001
Hydroxylysine	Hylys	2.28±0.08	2.26±0.06	2.37±0.15	0.396
Ornithine	Orn	28.91±1.45^c^	235.76±21.00^b^	295.83±28.88^a^	<0.001
Lysine	Lys	43.23±3.89	42.92±3.97	34.18±4.80	0.066
1-methylhistidine	1Mehis	13.61±1.17^a^	3.54±0.37^b^	11.61±0.81^a^	<0.001
Histidine	His	35.23±3.26^b^	49.51±4.33^a^	26.63±7.60^b^	0.006
Arginie	Arg	790.85±40.93^a^	11.86±1.50^c^	87.62±26.26^b^	<0.001
4-Hydroxyproline	Hypro	13.28±1.14^a^	6.86±1.56^b^	13.32±1.28^a^	0.002
Proline	Pro	15.43±2.67^b^	39.30±3.37^a^	11.67±2.31^b^	<0.001

Each value is expressed as mean ± SD (*n* = 9). The different lowercase shows significant difference (*P* < 0.05).

Different cooking methods resulted in different FFAs contents in crayfish meat. A total of 13 FAAs were higher in QM meat than in SG, including P-Ser, Tau, Asp, Gly, Ala, Cit, a-ABA, Val, EOHNH2, Orn, 1Mehis, Arg, and Hypro (*P* < 0.05). Conversely, six FAAs in QM lower than SG, including Thr, Tyr, Phe, b-AiBA, His, and Pro (*P* < 0.05). Gly is associated with a sweet taste (41), had the highest in QM at 146.47 mg/100 g. The Tau, Met and Tyr are bitter amino acids (42), and they were lower in QM and SG, corresponding to a decreased bitterness in SG and QM that detect by E-tongue. Valine, which decreased in both SG and QM (*P* < 0.05), exhibited intermediate taste qualities, with the taste thresholds of valine peptides depending on the presence of hydrophobic amino acid residues (43). Additionally, glutamate, a major contributor to umami flavor (44), was significantly higher in SG and QM compared with the CON (*P* < 0.05).

**FIGURE 5:**
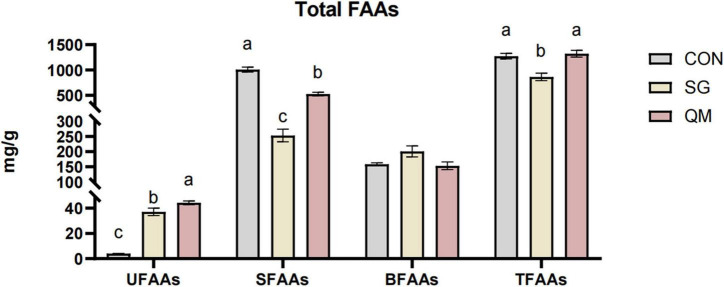
Total FAAs in crayfish meat processed by different methods. Typical umami amino acids (UFAAs), the total of Asp and Glu; sweet amino acids (SFAAs), the total of Thr, Ser, Gly, Ala, Arg, and Pro; bitter amino acids (BFAAs), the total of Val, Met, Ile, Leu, Phe, Tyr, Lys, and His TFAAs, the total free amino acids (TFAAs). Each value is expressed as mean ± SD (*n* = 9). The different lowercase letters indicate significant differences (*P* < 0.05).

Both stewing and quantitative marinating increased the levels of UFAAs compared with CON (*P* < 0.05, Figure 5 and Supplementary Table 3), and the umami amino acids in QM were increased by 19.47%. The UFAAs in QM and SG were 44.24 ± 1.48 mg/100 g and 37.04 ± 2.96 mg/100 g, respectively, much higher than those in CON (3.98 ± 0.22 mg/100 g, *P* < 0.05). In aquatic products, Asp and Glu have strong synergistic effects with umami nucleotides (45, 46). Crayfish meat in SG and QM had lower SFAAs compared with CON (*P* < 0.05). Compared with SG, total amino acids in QM were increased by 52.97%. Though, quantitative marinating processing did not change the TFAAs contents and stewing decreased total FAAs compared with CON, and BFAAs of each group were not significantly affected (*P* > 0.05). However, the composition and profiles of amino acids and their derivatives changed significantly (Table 1). When cooking crayfish, FAA content depends on processing temperature, with deep frying at 190°C resulting in the highest TFAA content in crayfish meat (10). FAAs contents are also related to processing methods. In marine fish meat, roasting increased the UFAAs contents compared with boiling and fry (47). Processing and cooking methods affect FAAs contents by modulating protein denaturation, with all FAAs contributing to the complex taste.

### 3.4 5′-nucleotides

**FIGURE 6:**
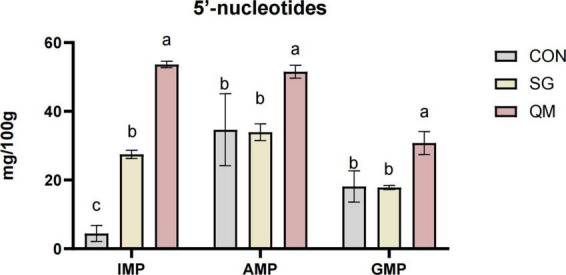
The effect of different processing methods on crayfish meat 5′-nucleotides contents including GMP, guanosine monophosphate, IMP, inosine monophosphate, and AMP, adenosine monophosphate. Each value is expressed as mean ± SD (*n* = 9). The different lowercase letters indicate significant differences (*P* < 0.05).

The flavor 5′-nucleotides, including GMP, AMP, and IMP, can synergistically enhance umami when combined with flavor amino acids. Figure 6 and Supplementary Table 4 presents the effects of different processing methods on the nucleotide content in crayfish meat. Crayfish meat in QM exhibited significantly higher levels of IMP, AMP, and GMP compared with CON and SG (*P* < 0.05). Stewing significantly increased the IMP content compared with the control (*P* < 0.05) but had no effect on AMP and GMP levels. IMP is a crucial component in producing the umami flavor in crayfish meat, functioning as an intermediate metabolite in amino acid biosynthesis and metabolism (48). Similarly, GMP is recognized for significantly boosting both the umami and the meaty flavors (49). Thus, it can be concluded that the synergistic effect of flavor 5′-nucleotides and umami amino acids improves the umami taste of crayfish meat during quantitative marinating process.

### 3.5 Volatile organic compounds

**FIGURE 7:**
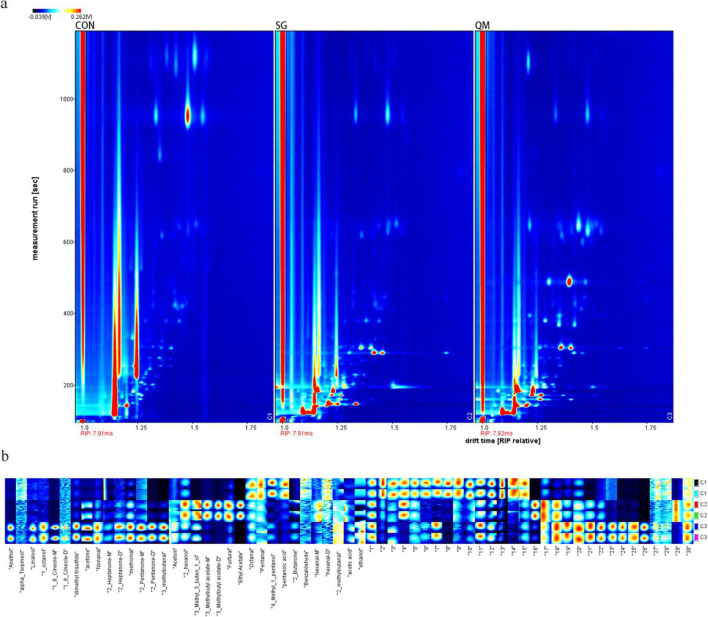
HS-GC-IMS analysis of crayfish meat. **(a)** HS-GC-IMS 2D spectrum of volatile compounds from crayfish meat. **(b)** Aroma fingerprint of crayfish samples. C1 stands for CON group; C2 stands for SG; and C3 stands for QM.

The HS-GC-IMS was performed to determine VOCs in crayfish meat processing by different methods. After data processing, two-dimensional spectrum images were generated (Figure 7a), where the ordinate represents retention time, and the abscissa denotes drift time. The red vertical line on the left represents the reactive ion peak (RIP), with a drift time of 7.9-7.92 ms. The retention time mainly located below 1,000 ms. Each red dot represents a volatile compound, with higher redness, the higher concentration. A total of 32 VOCs were identified (Supplementary Table 5). Due to varying concentrations, specific compounds can form dimers (50). As shown in Figure 6a, there were more VOCs highlights in SG and QM, and SG exhibited similar patterns to QM due to the use of the same spice system.

The LAV software was utilized to generate peak signal graphs, which served as the aroma fingerprint of the crayfish samples (Figure 7b), allowing for a comprehensive comparison of VOC levels. In Figure 7b, it is evident that Octanal, Pentanal, 4-Methyl-1-pentanol, and pentanoic acid were abundant in the control sample. Octanal and Pentanal were reported to have “fruity” taste (51); however, at high concentrations, they may produce an unpleasant odor (52). Pentanoic acid, an organic acid with an unpleasant odor, is commonly found in cooked pork (53). The QM sample was rich in several aroma compounds, including anethol and linalool, which are found in anise and other monocotyledonous and dicotyledonous plants and positively contribute to the flavor of meat (54, 55). This suggests that the quantitative marinating method enhances the interaction of spices with crayfish meat. Takakura et al. (56) identified 1-octanol as a critical aromatic compound in pork soup (56), which was also present in high levels in QM. Furthermore, 1,8-cineole, a terpenoids, is reported to significantly contribute to the aroma of porcine frankfurters (57). Nonanal, a typical aldehyde flavor compound, is commonly found in dry-cured meat (58). In SG sample, six VOCs were present at high levels, less than QM. 2-Hexanol, furfural, and ethyl acetate are known to contribute to meat flavors formed via the Maillard reaction (59). In summary, the quantitative marinating processing method enhances the aromatic compound profile of crayfish meat.

### 3.6 Biogenic amines

**FIGURE 8:**
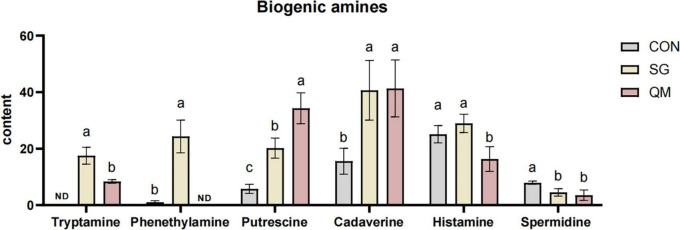
BAs contents in crayfish meat. Each value is expressed as mean ± SD (*n* = 9). The different lowercase letters indicate significant differences (*P* < 0.05).

BAs are present at varying concentrations in meat and meat products, and their levels can serve as indicators for assessing the quality and safety of these products available on the market (60). Our previous study identified several spoilage bacteria that proliferated rapidly, mainly including *Enterococcus*, *Bacillus*, *Lactobacillus*, *Leuconostoc*, and *Weissella* (61). Many of these bacteria are present in raw materials and can be introduced through contamination before, during, or after processing (62). Consequently, we analyzed the levels of aromatic amines after 7 days of refrigeration at 4°C. Six BAs were detected in crayfish meat (Figure 8 and Supplementary Table 6). Compared with CON, crayfish meat from SG and QM showed increased levels of tryptamine, putrescine, and cadaverine (*P* < 0.05). SG meat also had higher levels of phenethylamine than CON, which was not detected in QM (*P* < 0.05). The presence of these bacteria in meat can be influenced by various factors, including the pH, chemical composition, handling and manufacturing processes, as well as the temperature and duration of storage (62). For aquatic products, BAs are primarily produced during storage or processing, particularly before crayfish enter the cold chain. There is a significant difference in BAs content between live and dead crayfish, which affects BAs content after processing. Besides, different processing methods lead to varying sterilization efficiencies, which also influence the formation of BAs in crayfish meat during storage subsequently (63). For instance, microwave treatment 95°C for 15 min of precooked seasoned crayfish meat can reduce both the total viable count and BAs level after 3 days of storage at room temperature (64). However, previous study mainly focused on monitoring methods for BAs, bacteria-derived BAs and sterilization methods for crayfish meat (65, 66). In this study, the higher levels of FAAs and volatile compounds in meat could associated with an increased tendency for BAs formation. Interestingly, SG and QM had a lower Spermidine after 7 days storage (*P* < 0.05). This results might because some spices in meat had a strong inhibitory effect on spermidine (67). Further validation is needed in future studies to assess BAs contents and microorganism growth.

## 4 Conclusion

In this study, we investigated a new processing strategy, QM, for crayfish meat. The crayfish meat processing by QM exhibited higher brightness, redness, and yellowness values, as well as higher hardness, gumminess and chewiness for tightly arranged muscle fiber in microstructure. According to E-tongue analysis, quantitative marinating reduced bitterness and astringency in the crayfish. For flavor and order compounds analysis, meat in QM had higher levels of sweet free amino acids, GMP, AMP, and IMP. Furthermore, QM methods for crayfish got more VOCs such as anethol, linalool and 1-octanol, and relatively lower biogenic amines than stewing method. Overall, quantitative marinating appears to be a more favorable method for preserving meat quality and flavor in crayfish.

## Data Availability

The raw data supporting the conclusions of this article will be made available by the authors, without undue reservation.
