# Supplementary material for: Effects of quantitative marinating on meat quality, biogenic amines, and flavor compounds in crayfish meat
Source: Front Nutr. 2025 Apr 10;12:1573987. doi: 10.3389/fnut.2025.1573987 (PMC12018247; doi:10.3389/fnut.2025.1573987)
Supplement: Supplementary file 1 [file Table_1.docx]

**Table S1 Meat color profiles of crayfish meat processed by different methods**

| **Item** | CON | SG | QM | *P* - value |
| --- | --- | --- | --- | --- |
| L* | 16.25±3.87^c^ | 30.4±5.19^b^ | 42.98±3.26^a^ | <0.001 |
| a* | 14.45±3.3^b^ | 16.76±3.91^b^ | 25.83±1.71^a^ | <0.001 |
| b* | 13.2±2.59^c^ | 16.44±3.45^b^ | 19.84±1.81^a^ | <0.001 |

L*, lightness, a* redness, b* yellowness

The data were expressed as the mean ± standard deviation (mean ± SD ).

In the same row, values with different small letter superscripts mean a significant difference (p < 0.05), the same as in the following.

**Table S2 Texture profiles of crayfish meat processed by different methods**

| Item | CON | SG | QM | *P* - value |
| --- | --- | --- | --- | --- |
| Hardness 1^st^ /g | 141.58±12.6^b^ | 151.08±7.96^b^ | 234.33±15.98^a^ | <0.001 |
| Hardness 2^nd^ /g | 144.4±24.22^b^ | 139.92±6.98^b^ | 215.83±18.95^a^ | <0.001 |
| Gumminess/g | 113.45±11.48^b^ | 116.12±5.66^b^ | 178.78±11.24^a^ | <0.001 |
| Cohesiveness/g | 0.402±0.024 | 0.400±0.037 | 0.388±0.01 | 0.641 |
| Resilience /mm | 1.59±0.07 | 1.6±0.05 | 1.63±0.09 | 0.632 |
| Chewiness /mJ | 1.77±0.19^b^ | 1.79±0.11^b^ | 2.86±0.26^a^ | <0.001 |
| Adhesiveness /g | 1.83±0.26 | 1.75±0.42 | 2.17±0.41 | 0.152 |

The data were expressed as the mean ± standard deviation (mean ± SD ).

In the same row, values with different small letter superscripts mean a significant difference (p < 0.05), the same as in the following.

**Table S3 Total FAAs in crayfish meat processed by different methods**

| Item | CON | SG | QM | *P* - value |
| --- | --- | --- | --- | --- |
| UFAAs (mg/g) | 3.98±0.22^c^ | 37.0±3.0^b^ | 44.3±1.5^a^ | <0.001 |
| SFAAs (mg/g) | 1006.2±49.6^a^ | 253.4±20.9^c^ | 524.9±34.3^b^ | <0.001 |
| BFAAs (mg/g) | 158.8±4.6 | 200.9±18.1 | 153.3±12.6 | 0.108 |
| TFAAs (mg/g) | 1273.7±53.8^a^ | 863.5±74.7^b^ | 1320.9±67.4^a^ | <0.001 |

The data were expressed as the mean ± standard deviation (mean ± SD ).

In the same row, values with different small letter superscripts mean a significant difference (p < 0.05), the same as in the following.

**Table S4 The 5’-nucleotides in crayfish meat processed by different methods**

| Item | CON | SG | QM | *P* - value |
| --- | --- | --- | --- | --- |
| GMP(mg/100g) | 1.81±0.45^b^ | 1.78±0.06^b^ | 3.08±0.34^a^ | 0.004 |
| IMP(mg/100g) | 4.44±2.32^c^ | 27.48±1.19^b^ | 53.65±0.92^a^ | <0.001 |
| AMP(mg/100g) | 34.65±10.48^b^ | 33.92±2.46^b^ | 51.55±1.87^a^ | 0.023 |

The data were expressed as the mean ± standard deviation (mean ± SD ).

In the same row, values with different small letter superscripts mean a significant difference (p < 0.05), the same as in the following.

**Table S5 Total volatile organic compounds detected by GC-iMAS**

| count | compound | CAS | Formula | MW | RI | Rt[sec] | Dt[RIP rel] |
| --- | --- | --- | --- | --- | --- | --- | --- |
| 1 | Anethol | C104461 | C10H12O | 148.2 | 1309.9 | 1100.171 | 1.2115 |
| 2 | Linalool | C78706 | C10H18O | 154.3 | 1102.3 | 644.606 | 1.2162 |
| 3 | Nonanal | C124196 | C9H18O | 142.2 | 1096.2 | 631.306 | 1.482 |
| 4 | 1-octanol | C111875 | C8H18O | 130.2 | 1080.9 | 597.733 | 1.455 |
| 5 | 1,8-Cineole | C470826 | C10H18O | 154.3 | 1029.3 | 489.959 | 1.3053 |
| 6 | 1,8-Cineole | C470826 | C10H18O | 154.3 | 1027.7 | 486.835 | 1.7238 |
| 7 | Octanal | C124130 | C8H16O | 128.2 | 994.2 | 427.017 | 1.4234 |
| 8 | dimethyl trisulfide | C3658808 | C2H6S3 | 126.3 | 961.3 | 379.014 | 1.2888 |
| 9 | 2-Heptanone | C110430 | C7H14O | 114.2 | 892.5 | 304.582 | 1.2619 |
| 10 | 2-Heptanone | C110430 | C7H14O | 114.2 | 892 | 304.159 | 1.6324 |
| 11 | methional | C3268493 | C4H8OS | 104.2 | 890.6 | 302.933 | 1.4054 |
| 12 | 4-Methyl-1-pentanol | C626891 | C6H14O | 102.2 | 859.2 | 276.878 | 1.3503 |
| 13 | 2-hexanol | C626937 | C6H14O | 102.2 | 778.1 | 221.089 | 1.2804 |
| 14 | 3-Methyl-3-buten-1-ol | C763326 | C5H10O | 86.1 | 732.2 | 194.114 | 1.5034 |
| 15 | 2-Pentanone | C107879 | C5H10O | 86.1 | 684.3 | 171.737 | 1.3704 |
| 16 | 2-Pentanone | C107879 | C5H10O | 86.1 | 686.7 | 172.657 | 1.1206 |
| 17 | 3-methylbutanal | C590863 | C5H10O | 86.1 | 649.9 | 159.782 | 1.404 |
| 18 | acetone | C67641 | C3H6O | 58.1 | 507.3 | 122.079 | 1.1152 |
| 19 | 2-Butanone | C78933 | C4H8O | 72.1 | 585.9 | 142.31 | 1.2428 |
| 20 | pentanoic acid | C109524 | C5H10O2 | 102.1 | 922.6 | 333.589 | 1.2271 |
| 21 | Pentanal | C110623 | C5H10O | 86.1 | 674.5 | 168.016 | 1.1824 |
| 22 | 3-Methylbutyl acetate | C123922 | C7H14O2 | 130.2 | 875.4 | 289.945 | 1.7472 |
| 23 | 3-Methylbutyl acetate | C123922 | C7H14O2 | 130.2 | 877.3 | 291.531 | 1.3059 |
| 24 | Furfural | C98011 | C5H4O2 | 96.1 | 791.8 | 229.727 | 1.3357 |
| 25 | Ethyl Acetate | C141786 | C4H8O2 | 88.1 | 605 | 147.251 | 1.3357 |
| 26 | alpha-Terpineol | C98555 | C10H18O | 154.3 | 1171.5 | 796.483 | 1.2169 |
| 27 | Benzaldehyde | C100527 | C7H6O | 106.1 | 961.9 | 379.731 | 1.467 |
| 28 | hexanal | C66251 | C6H12O | 100.2 | 790 | 228.574 | 1.5596 |
| 29 | hexanal | C66251 | C6H12O | 100.2 | 792.6 | 230.232 | 1.2732 |
| 30 | Acetoin | C513860 | C4H8O2 | 88.1 | 732.3 | 194.178 | 1.2475 |
| 31 | 2-methylbutanal | C96173 | C5H10O | 86.1 | 660 | 163 | 1.1717 |
| 32 | acetic acid | C64197 | C2H4O2 | 60.1 | 590.5 | 143.483 | 1.0518 |
| 33 | ethanol | C64175 | C2H6O | 46.1 | 489.1 | 117.407 | 1.0437 |

**Table S6 BAs contents in crayfish meat**

| Item | CON | SG | QM | *P* - value |
| --- | --- | --- | --- | --- |
| Tryptamine | nd | 17.55±2.96^a^ | 8.45±0.64^b^ | 0.007 |
| Phenethylamine | 1.01±0.59^b^ | 24.36±5.77^a^ | nd | 0.002 |
| Putrescine | 5.84±1.6^c^ | 20.22±3.55^b^ | 34.34±5.46^a^ | <0.001 |
| Cadaverine | 15.62±4.57^b^ | 40.69±10.54^a^ | 41.33±10.08^a^ | 0.019 |
| Histamine | 25.15±3.09^a^ | 28.97±3.27^a^ | 16.39±4.34^b^ | 0.014 |
| Spermidine | 0.8±0.06^a^ | 0.46±0.13^b^ | 0.36±0.19^b^ | 0.017 |

The data were expressed as the mean ± standard deviation (mean ± SD ).

In the same row, values with different small letter superscripts mean a significant difference (p < 0.05), the same as in the following.
